# Development and Optimization of a Miniaturized Western Blot-Based Screening Platform to Identify Regulators of Post-Translational Modifications

**DOI:** 10.3390/ht8020015

**Published:** 2019-06-03

**Authors:** Florencia Villafañez, Vanesa Gottifredi, Gastón Soria

**Affiliations:** 1 Centro de Investigaciones en Bioquímica Clínica e Inmunología, CIBICI-CONICET, Córdoba X5000, Argentina; florenciavillafaez@yahoo.com.ar; 2 Departamento de Bioquímica Clínica, Facultad de Ciencias Químicas, Universidad Nacional de Córdoba, Córdoba X5000, Argentina; 3 Fundación Instituto Leloir_Instituto de Investigaciones Bioquímicas de Buenos Aires, CONICET, Buenos Aires C1405BWE, Argentina; vgottifredi@gmail.com

**Keywords:** post-translational modification, western blot, screening

## Abstract

Post-translational modifications (PTMs) are fundamental traits of protein functionality and their study has been addressed using several approaches over the past years. However, screening methods developed to detect regulators of PTMs imply many challenges and are usually based on expensive techniques. Herein, we described the development and optimization of a western blot-based platform for identification of regulators of a specific PTM—mono-ubiquitylation of proliferating cell nuclear antigen (PCNA). This cell-based method does not require specific equipment, apart from the basic western blot (WB) devices and minor accessories, which are accessible for most research labs. The modifications introduced to the classical WB protocol allow the performance of PTM analysis from a single well of a 96-well plate with minimal sample manipulation and low intra- and inter-plate variability, making this method ideal to screen arrayed compound libraries in a 96-well format. As such, our experimental pipeline provides the proof of concept to design small screenings of PTM regulators by improving the quantitative accuracy and throughput capacity of classical western blots.

## 1. Introduction

Post-translational modifications (PTMs) are key marks of proteins that are introduced after their biosynthesis and regulate localization, activity and final functionality. Common modifications are phosphorylation, glycosylation, methylation, acetylation, amidation, ubiquitylation, carboxylation, disulfide bond, hydroxylation, nitrosylation and sumoylation. PTMs can also control protein–protein interactions and are thus able to modulate not only the function of the protein but also its interacting partners [1]. As an additional layer of complexity, more than one PTM can be present in the same protein. Moreover, some PTMs are reversible and their regulation responds to changes in the cellular environment as well as exogenous stimuli, rendering their study useful for the detection of aberrant cellular pathways, biomarker development and ultimately as targets for precision medicine therapeutics [2,3,4].

A variety of experimental methods have been used to detect PTMs, such as mass-spectrometry (MS), reverse phase protein arrays (RPA) and western blot (WB) [5,6,7]. MS-based methods are powerful techniques used to detect PTMs substrates and to map PTMs sites. The basic protocol involves the digestion of the protein lysate of interest by a specific protease (usually trypsin) and an enrichment step for the specific PTM and analysis by LC-MS/MS. Following acquisition of data, computational algorithms are used for peptide and protein identification as well as their relative quantification. RPA involves a platform that immobilizes cell lysates in small spots and enables the quantification of PTMs of preselected proteins by antibodies that specifically recognize the PTM of interest. WB requires the separation of protein from complex cellular lysates according to their molecular weight within an acrylamide gel, and their posterior transfer into a membrane for blotting with primary and secondary antibodies for the selected PTMs. 

Sample complexity is a general disadvantage in all the previously mentioned methodologies. MS-based methods fraction the peptides to reduce this issue before the LC-MS/MS, but this technique decreases sample throughput and increases costs. MS-based methods can detect and quantify with high precision several PTMs, while certain classes of modifications, such as glycosylation with large glycans and nucleotide modifications, can be challenging to detect using this technique. Other limitations to be considered in MS are the abundance of substrate proteins, the co-occurrence of different or equal PTMs in the same protein and the associated stability due to the partial protein decomposition during the analysis [8]. Regarding RPA assays, they are restrained by the unavailability of highly-specific antibodies validated for this technique [9]. Such disadvantage also applies for WB experiments used to study multiple PTMs. 

Even though many aspects of the aforementioned methodologies have significantly improved in the last years, the design of high-throughput screenings (HTS) to identify modulators of PTM still lags behind. Furthermore, several challenges arise in relation to the feasibility to perform screenings for the complex repertoire of PTMs. More importantly, such experimental designs are expensive and unaffordable for many members of the research community, especially in low- and middle-income countries. Most HTS performed to identify PTM regulators are conducted using in vitro-based assays, such as the AlphaScreen technology, Förster resonance energy transfer (FRET)-based platforms and quenching resonance energy transfer (QRET) [10,11]. These types of platforms require an intricate set-up that entails the generation and purification of suitable substrates, and the tagging of fluorescent proteins [12]. While efforts towards a universal HTS assay to enable direct detection of a wide variety of PTMs are undergoing , most used technologies still rely on in vitro-based assays [13]. In all cases, such complex and expensive approaches are the first step of the identification of PTMs, since all the aforementioned methods need to be followed by the validation of individual results. 

A different approach that is increasingly used by researchers is the in situ proximity ligation assay (PLA) reported by Leuchowius et al. [14]. Their goal was to identify compounds that inhibited the platelet-derived growth factor (PDGF) receptor signaling pathway in primary human fibroblast. In order to automatize the examination of PLA results, they used high content analysis with a Cellomics ArrayScan II automated fluorescence microscope. Though this is a clear step forward in the design of HTS for the identification of PTM inhibitors, it has two major limitations. It involves expensive equipment and retains the central limitation shared by all immuno-based assays—the requirement of antibodies that can efficiently detect the PTM in its native conformation.

The outstanding variety of PTMs poses a methodological and technological challenge to studies aimed at identifying their potential regulators. Such a limitation is particularly relevant for those PTMs for which the only types of available antibodies are against the denaturized form of the protein. In such cases, researchers still need to rely upon classic methods such as WB or dot-blot, which impose serious limitations to the throughput capacity of the screenings. Herein, we describe the development and optimization of a western blot-based screening platform for the study of a specific PTM—mono-ubiquitylation of proliferating cell nuclear antigen (PCNA). This platform can easily be adapted to the evaluation of other PTMs and presents a number of advantages. It does not require specific equipment apart from the standard WB devices available in every laboratory, and it involves a single step preparation of samples, requiring only the sample from a single well from a 96-well plate. Together, such modifications of the WB technology allow a significant increase in the throughput capacity and robustness of the method. The importance of this work lies on the several methodological modifications that we introduced to the classical WB protocol, which should help other research groups that wish to establish small screening projects with arrayed compound libraries.

## 2. Materials and Methods

### 2.1. Antibodies

The primary antibodies used were α-ubiquityl-PCNA (D5C7P; Cat# 13439) and α-PCNA (PC-10; Cat# 2586). The secondary antibodies used were goat α-mouse IRDye 680RD (Cat# P/N 925-68070) and goat α-rabbit IRDye 800CW (Cat# P/N 925-32211) from LI-COR Biosciences.

### 2.2. Cell Culture, Transfections and UV Irradiation

U2OS were acquired from ATCC and cultured in DMEM medium (Thermo Fisher Scientific, MA, USA) supplemented with 5% FBS (GIBCO). Global UV irradiation with 15J/m^2^ was performed as previously described [15]. The U2OS cell line was negative for mycoplasma contamination by multiplex PCR.

For the experiments, cells were first washed with PBS, incubated with trypsin (Cat#15400054, Thermo Fisher Scientific) at 37 °C for 2 min and then harvested in DMEM medium with 5% FBS. Cells were then manually counted and plated by manual pipette or liquid handling pipette.

### 2.3. Protein Analysis

For direct western blot analysis, samples were washed once with PBS, resuspended and lysed in one single step in commercial Laemmli buffer (BioRad, CA, USA) containing the reducing agent 2-mercaptoethanol (355 mM, Sigma, MI, USA) and benzonase nuclease (250 U/ul, Santa Cruz, CAS 9025-65-4). No protease inhibitors are used for lysis. For experiments of proof of concept, cells in a 96-multiwell plate were washed once with PBS and then lysed directly in the plate with 30 µL of commercial Laemmli buffer with benzonase per well. 

Gels at 12% acrylamide:bisacrylamide (29:1) were casted using a mini-protean 3 multi-casting chamber (BioRad). Samples were loaded into gels directly from the 96-multiwell plate and resolved in a mini-protean tetra vertical electrophoresis cell (BioRad) and transferred to PVDF membranes (Roche). Transfer was performed using a semidry system from BioRad. 

Membrane blocking was performed with a solution containing tris-buffered saline (TBS), Tween 20 at 0.1% and 5% non-fat milk. Primary antibodies were used at a concentration of 1:1000 (α-ubiquityl-PCNA) and 1:2000 (PCNA). Secondary antibodies were used at a concentration of 1:10000. Antibodies were diluted in TBS-Tween at 0.1%.

The detection and quantification of the near-infrared fluorescence on the membranes was performed with an Odyssey CLx System (LI-COR Biosciences) through the Image Studio Software. Images were acquired on auto intensity at high resolution with both channels (700 and 800) simultaneously.

### 2.4. Automatic Capture of Brightfield Images

Cells seeded in a 96-multiwell plate were imaged automatically in a Leica DMI8 microscope. Parameters set for the automatic capture are as follows: fixed starting z-position of 4130; autofocus with 15 captures of 200 µm apart from each other and then selection of the best image according to contrast. 

### 2.5. Statistical Analysis

All samples were plated as duplicate or triplicate. All experiments were performed two or three times. Statistical analysis and plotting were performed using GraphPad Prism 5.0 (GraphPad Software, La Jolla, CA, USA).

## 3. Results

### 3.1. Comparative Analysis of Techniques Available for the Set-Up of MTS-Based Studies of PTMs

The main goal of this project was to set up a low- to medium-throughput screening (MTS) assay aimed at identifying regulators of PTMs using the technology available in standard academic laboratories. While we focused our screening on a single PTM modification, our method and troubleshooting experience can be used as a tutorial to study any PTM on any protein. We describe the optimization of the method and the challenges we faced while setting up the WB-based MTS assay in our laboratory. Our aim was to identify inhibitors of the mono-ubiquitylation of PCNA (ubi-PCNA), a PTM that plays an important role in translesion DNA synthesis (TLS). It was key for the success of this set-up to count with an antibody that specifically detects the mono-ubiquitylated form of PCNA and another that detects total PCNA (modified and unmodified). 

As an initial approach, we evaluated the possibility to perform in-cell western blot (IC-WB), because of the advantages that this technology has in terms of robustness and throughput capacity for screening purposes. Such a technique can be summarized in the following few steps: (A) seeding of cells in a 96-multiwell format; (B) treatment of cells with potential PTM inhibitors; (1) fixation with PFA; (2) permeabilization with methanol; (3) blocking; (4) probing with primary and (5) secondary antibodies (Figure S1A). The analysis of samples was performed in the Odyssey CLx System (LI-COR Biosciences) using the associated Image Studio Software (Figure S1B). The IC-WB has many advantages—as it can be performed in a 96-multiwell format, it may be easily automated with liquid handling systems. Depending on the linear range of each antibody, it could be used for quantification analysis. Importantly, it also allows the simultaneous detection of up to two different antigens per well, being easily applicable to HTS platforms that detect proteins in a relevant cellular context. Unfortunately, this method was not satisfactory in our case due to its failure to detect the expected increase in the levels of ubi-PCNA when comparing non-irradiated (-UV) vs. irradiated (+UV) samples (Figure S1B). The expected differential levels of PCNA ubiquitylation were detected by the same ubi-PCNA antibody when using the WB approach (Figure S1C). We hence speculate that in the IC-WB conditions, in which neither size separation nor denaturation are applied, the ubi-PCNA antibody may non-specifically interact with one or many proteins, increasing the background of the immunostaining and precluding the detection of ubi-PCNA.

**Figure 1:**
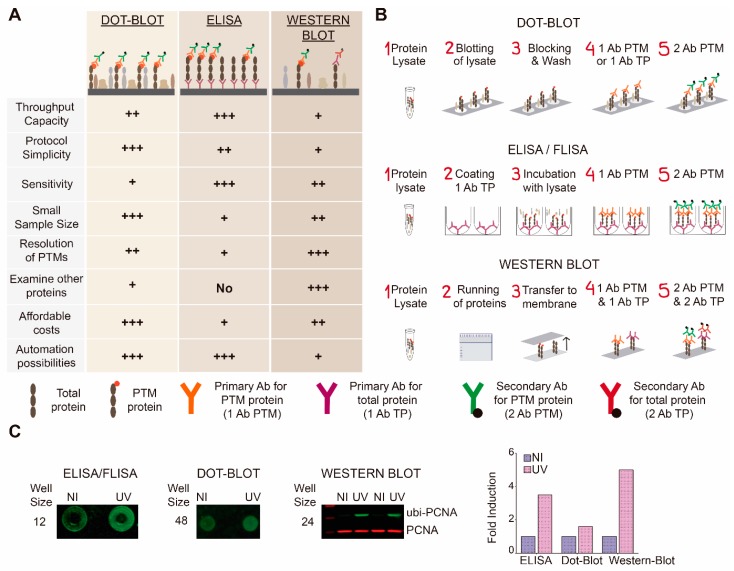
Study of techniques available for detection of post-translational modifications of proteins in a medium-throughput screening (MTS) setup. (**A**) Analysis of advantages and disadvantages presented by dot-blot, enzyme-linked immunoabsorbent assay (ELISA) or fluorophore-linked immunosorbant assay (FLISA) and western blot (WB) regarding post-translational modifications (PTM) detection (other proteins in the lysate are represented with figures of different shapes and colors); (**B**) General experimental pipeline of each technique; (**C**) Representative images depicting the detection of mono-ubiquitylation of proliferating cell nuclear antigen (ubi-PCNA) by FLISA, dot-blot and WB and quantification of fold induction between non-irradiated (NI) and UV irradiated (UV 15 J/m^2^) samples displayed by each technique.

Then, the next logical alternative was to assess the enzyme-linked immunoabsorbent assay (ELISA) technology, which is another antibody-based method developed to detect an antigen in a sample in both a quantitative and qualitative manner. The basic ELISA protocol involves the following steps: (A) seeding of cells in a 96-multiwell format; (B) treatment of cells with potential PTM inhibitors; (1) immobilization of the primary antibody on a solid support, e.g., a 96-multiwell plate, and wash of unbound antibodies; (2) addition of a soluble protein sample, e.g., a lysate obtained from cells and washing cycles; (3) addition of a primary antibody against the PTM and a wash of unbound primary antibodies; (4) incubation with a secondary antibody and a final wash of unbound secondary antibodies (Figure 1B). The detection of fluorescence of the secondary antibodies is performed with Odyssey CLx equipment. ELISA protocols have few advantages. For instance, it is easy scalable for high-throughput screening applications and it can be quantitative, mainly depending on the dynamic range of the antibodies used. As it is usually performed in a 96-multiwell format, it can also be easily automated with liquid handling systems (Figure 1A). However, ELISA approaches also have a number of limitations which are intrinsic to the nature of the assay, the most notorious one being its inability to detect more than a single target (PTM or protein). Moreover, the targeted PTM needs to be robustly induced to grant detection by this method. Larger amounts of samples can be used to overcome such limitation. In fact, when evaluating ubi-PCNA induction, we were forced to increase the sample size threefold in conditions of strong PTM induction triggered by UV irradiation in order to be able to detect such a PTM modification with the ELISA approach. Such a considerable increase in sample size would very much limit the screening throughput and, therefore, the ELISA technology was discarded (Figure 1C). 

The follow-up alternative to consider was the dot-blot assay, a simple methodology that at many steps resembles the classical WB. Briefly, dot-blot consist in the following steps: (A) seeding of cells in a multiwell format; (B) treatment of cells with potential PTM inhibitors; (1) cell lysis; (2) immobilization of proteins on a PVDF membrane; (3) blocking with bovine serum albumin (BSA) or other protein-rich solution; (4) probing with single primary and (5) secondary antibodies (Figure 1B). The analysis of samples is performed in the Odyssey Clx System (LI-COR Biosciences). This technique allows automation, since the lysate can be immobilized on PVDF in dots corresponding to a 48–96-multiwell format. Therefore, medium-throughput experiments are affordable and no special equipment is required. A major drawback of this method is that it does not separate proteins by size, and therefore the sequential analysis of several proteins in the same blot is highly difficult to set up and frequently unreliable (Figure 1A). Moreover, because samples are immobilized into very limited areas, the high concentration of multiple proteins in the same location increases the background of the immunostaining, leading to a considerable reduction of sensitivity. Consistently with such limitations, the detection of PCNA ubiquitylation induction after UV was weak in the dot-blot settings, probably as a consequence of high background signals (Figure 1C).

Lastly, we explored the classical western blot (WB) as a possible method for the screening of inhibitors of ubi-PCNA. WB is a technique regularly used for the analysis of proteins. It consists of three main steps: gel electrophoresis, membrane transfer and blotting and probing with antibodies (Figure 1B). While it has been recommended for low-throughput analysis, it offers more disadvantages than advantages when adapting it to screening platforms. The throughput capacity of WB-based assays is poor, since the amounts of sample required are difficult to scale down, and because sample preparation involves multiple tedious steps. Moreover, automation possibilities are limited (Figure 1A). Nonetheless, WB offers higher sensitivity than dot-blot due to the separation of proteins by molecular size, and also the possibility of simultaneously examining more than one protein simultaneously, or even after the screening is completed (Figure 1A). An initial evaluation of the WB-based approach revealed the ubi-PCNA provided high sensitivity and good linear range (not shown). Moreover, the WB MTS settings allowed simultaneous incubation with a PCNA antibody to detect total PCNA as a loading control for the PTM (Figure 1C).Therefore we decided to focus our efforts in improving the throughput capacity of this method and overcoming the previously mentioned disadvantages of this technique.

### 3.2. Optimization of the Throughput Screening Capacity of the Classical Western Blot Assay

**Figure 2:**
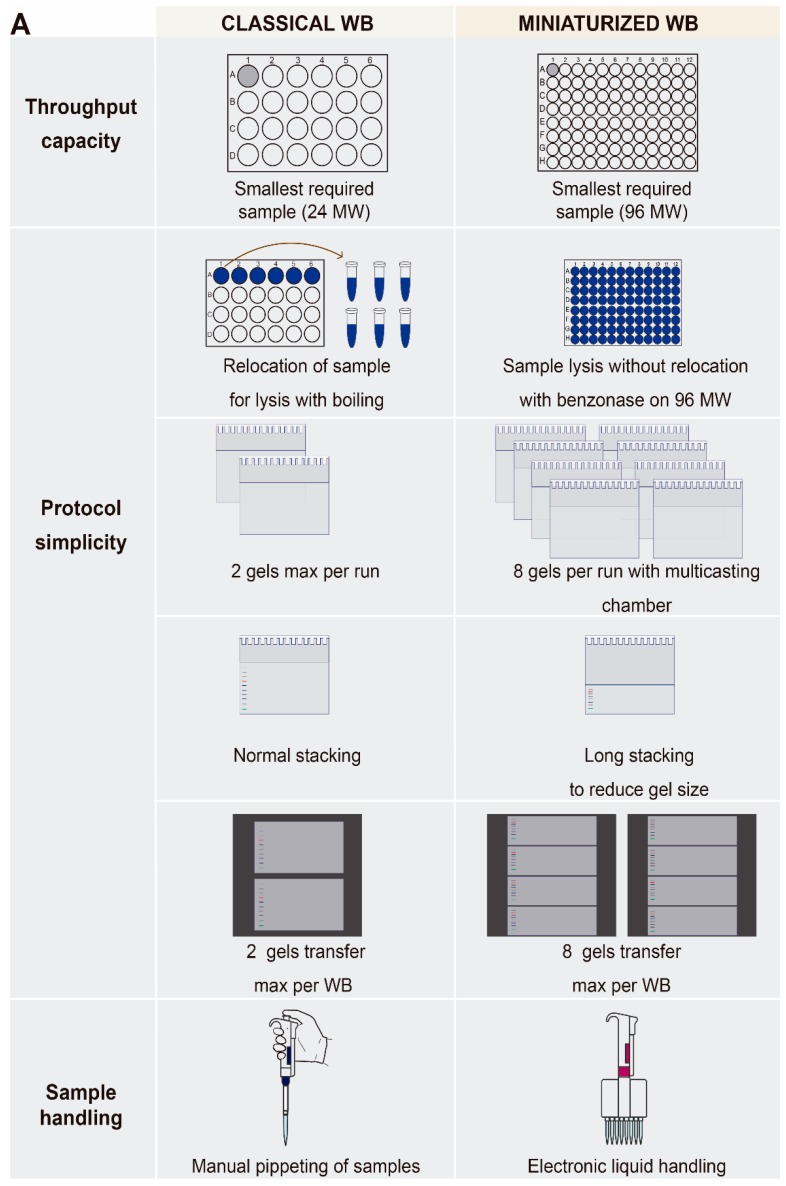
Characteristics of the optimization stage of the screening platform going from a classical WB towards a miniaturized WB focusing on three major areas: throughput capacity, protocol complexity and sample handling.

Two main issues were tackled in this optimization:reaching the smallest sample size (in our case a 96-multiwell (MW) plate) and reducing the sample manipulation in the screening plates (Figure 2). The WB protocol comprises a number of steps that imply sample relocation (Figure 1). More steps can increase manipulation mistakes, especially when dealing with a high number of samples within a screening setting. Initially, we aimed to limit the number of steps by avoiding sample relocation after cellular lysis and before gel loading. The sample relocation step is required for the denaturalization of proteins. The most commonly used technique for denaturalization of proteins is a boiling step of the sample, which is preceded by resuspension in loading buffer with SDS. Such denaturalization enables antibody recognition and loading of samples into the SDS acrylamide gel (Figure 2). In order to avoid the relocation of samples from MW plates to tubes, the boiling step should be eliminated. As an alternative method, we treated samples with benzonase, a nuclease from *Serratia marcescens* that degrades all forms of DNA and RNA [16,17]. This enzyme is effective across a number of conditions, such as different pH levels and detergent concentrations. Benzonase has also been known to enhance protein resolution in two-dimensional (2D) gel electrophoresis by removing any bound nucleic acids. We tested this enzyme at different concentrations and observed that the best resolution was achieved when it was added directly to the sample buffer at 1/1000 concentration (containing 1% LDS). The optimal incubation time was 30 min at 37 °C. Benzonase treatment immediately was followed by the loading of samples to gels directly from the screening plates (Figure 2). 

A second limitation of WB is the poor throughput associated with the limited number of gels that can be casted at the same time and the comparability between gels (length of stacking and separating gels). To reduce this variability, we recommend the use of a multicasting chamber such as the one offered by BioRad, which allows the casting of up to 12 gels simultaneously, increasing reproducibility (Figure 2). As we were not using precasted gels, the timing of polyacrylamide polymerization was taken into account. We concluded that TEMED concentration must be reduced in order to prevent acrylamide polymerization while pouring the solution into the multiple (eight) chambers used for our analysis. We also concluded that the resolution portion of gels (12% polyacrylamide) could be rather short, yet allowed for optimal separation of PCNA and ubi-PCNA (29 and 38kDa, respectively). While the standard length of the separating gels (approximately 5 cm for mini-gels) precluded simultaneous semidry transfer due to lack of space in the transfer device, a reduction in the size of separating gels enabled the transfer of up to four gels in each semi dry trans blot (Figure 2). 

As a final consideration, at every possible step, such as cell seeding, media removal and sample buffer addition, we recommend automation. For example, we found out that employing electronic liquid handling pipettes and a VIAFLO ASSIST pipetting arm remarkably reduced variability. By using the set-up described above, we reached a final screening capacity of 80 compounds tested within a single experiment conducted in a 96-multiwell format (Figure 2).

### 3.3. Proof of Concept Screening with Our Miniaturized WB-Based Platform: General Considerations

**Figure 3:**
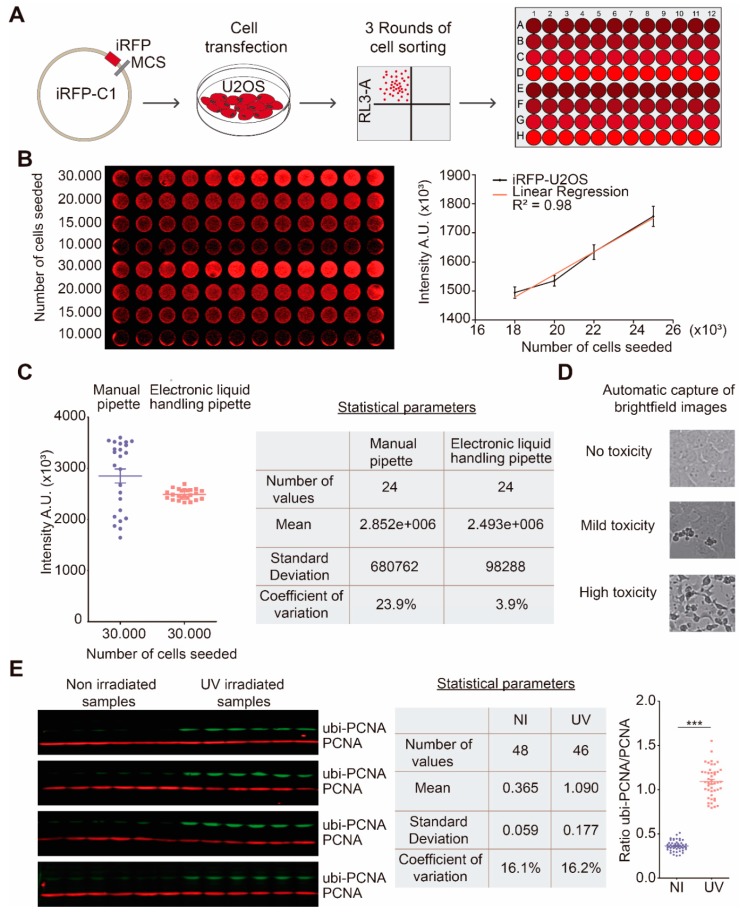
Quality controls and initial testing of the screening platform. (**A**) Development of stable cell line infrared fluorescent protein (iRFP)-U2OS by transfection of a iRFP-C1 plasmid and following cell sorting; (**B**) Assessment of the linear correlation between cell number seed and intensity detected by Odyssey equipment. The left image displays a 96-multiwell plate, where each column is an experimental replica for each condition (e.g., 12 replicas for 10,000 cells); (**C**) Variability and statistical parameters between different systems used for cell seeding; (**D**) Representative automatically-captured brightfield images depicting the range of toxicity that could be expected in the MTS. *(***E**) Proof of concept of the screening platform with non-irradiated and UV-irradiated samples (15 J/m^2^) and statistical parameters of each condition.

After optimization of the selected WB-based approach, we pursued the development of quality controls to monitor cell number, intra-well distribution of cells and edge effects prior to launching the actual screening experiments. To this end, we established a U2OS cell line expressing infrared fluorescent protein (iRFP) with the transfection of an iRFP-C1 plasmid and performed three sequential rounds of sorting. Such a number of rounds were required to ensure a population with high and homogenous expression of the plasmid (Figure 3A). Given the great range of fluorescent intensity provided by the LICOR CLx infrared scanner, there is a correlation between cell number and fluorescent intensity [18]. Such a cell line enabled us to estimate the number of cells before lysis directly on the 96 screening plates. This was possible because the emission wavelength of this fluorescent protein was 713 nm and the detector of this equipment was 700 nm (Figure 3B). Remarkably, in our stable cell line, and by comparing results with manual counting, we concluded that by scanning iRFP-C1 emission it is possible to estimate the total cell number in a range of 10,000 to 30,000 cells per well. In fact, within that range, there was a linear correlation between the number of cells seeded and the intensity detection, measured in arbitrary units (A.U) (Figure 3B). Above such cell numbers, the co-relation between manually counted cells and the fluorescence intensity was not linear (not shown). 

As mentioned previously, a great source of variability in the final cell number was encountered when evaluating different methods to seed the cells. When a manual system for sample manipulation was used, the final cell number measured in A.U associated with iRFP-C1 emission was notably dispersed, even when cells were seeded with high quality mechanical pipettes. On the contrary, when an electronic liquid handling system such as Integra Viaflow Assist was employed, the variability was minimized (Figure 3C). Such an ability to limit plating variation provided us with a simple quality control tool for our screening experiments. We could easily detect irregularities arising during cell seeding, e.g., mistakes in the number of plated cells or edge effects. Importantly, such experience allowed us to determine the acceptable range of variability in the intra-well distribution. When any of the aforementioned irregularities were detected, the experiment was immediately discarded, avoiding the futile utilization of compounds that were supposed to be screened, and preventing the accumulation of false positive/negative results. 

Since we were also interested in assessing the effect of each compound both on the PTM and on the cellular fitness, we incorporated an extra step in the screening protocol that allowed us to evaluate the general toxicity of each compound. To do so, we developed an automatic capture program for bright field images, taking one picture of each 96-well of the plate prior to sample lysis with a Leica DMI8 microscope equipped with a motorized stage. This allowed the identification of compounds which were either severely toxic at the concentration tested or that somehow affected cellular integrity (Figure 3D).

Our proof-of-concept screening using this platform with multiple replicas of non-irradiated and UV-irradiated conditions showed that the coefficient variance (CV%) is acceptable and lies below the one observed for a routine WB (Figure 3). 

## 4. Discussion

In this work we have approached the major limitations and challenges to study modulators of PTMs in a screening setup within an academic laboratory, with a focus on economically accessible methodologies adapted to available antibodies that detect the PTM of interest. Our conclusion is that when antibodies detect the PTMs in their native confirmation, methods such as in-cell western (ICW) provide an excellent compromise between sensitivity range, loading control capabilities, throughput capacity and small costs for small academic labs (Figure S1). However, a major drawback is encountered when the only available antibodies for the PTM of interest detect exclusively the PTM in the context of the denatured protein. Under these conditions, our detailed analysis of potential methods led us to the conclusion that the classical western blot is the most robust approach when considering sensitivity, reproducibility and sufficiently small sample size that proved compatible with a screening setup (Figure 1). Even though we managed to adapt the sample size to a single 96-well plate, the main challenge we faced was to decrease sample manipulation and relocation (Figure 2). Aside from the incorporation of multichannel electronic pipettes and a small robotic pipetting arm, the critical step we avoided was sample boiling and relocation from MW plates to tubes. This was possible by the addition of benzonase to the loading buffer, followed by a short incubation period (Figure 2). As such, we were able to perform cell seeding, drug addition, UV irradiation and sample lysis in the same 96-MW plate, and were also able to load the gels straight from the screening plates. This setup allowed us to screen 80 compounds per plate plus the untreated and positive controls (Figure 2). 

We also invested substantial efforts in improving the reproducibility, decreasing the CVs and establishing several quality controls before and after the addition of drugs (Figure 3). Automation was also central for the success of these controls. Aside from the initial cell counting prior to cell seeding in the 96-MW screening plates, we incorporated an additional step to assess cell number and distribution across the screening plates. This was possible using a stably expressed infrared fluorescent protein (iRFP), which we were able to scan prior to the addition of drugs within the same screening plates (Figure 3). We concluded that the lowest CVs and the more homogenous well-to-well cellular distribution were reached when a high number of cells per well are seeded (~25K) using a pipetting assisting arm (Figure 3). This proved to be a critical quality control that warranted the success of MTS experiments. 

Altogether, in this paper we present a series of strategies that may be employed by other research groups that want to perform small screenings for PTM modulators. Even though an additional setup would certainly be required if a different PTM and/or loading control are used, most of our experimental pipeline will remain similar if an MTS cell-based WB approach is considered. Our findings may be of importance, especially for academic labs with a low budget, which do not have expensive heavy equipment or HTS facilities available. Our detailed protocol should provide strategies to strikingly increase the throughput capacity of classical WB, reaching high levels of accuracy and reproducibility to successfully face small screening projects.

## Supplementary Materials

The following are available online at https://www.mdpi.com/2571-5135/8/2/15/s1, Figure S1: Experimental Analysis of IC-WB.
