# Supplementary material for: Development and Optimization of a Miniaturized Western Blot-Based Screening Platform to Identify Regulators of Post-Translational Modifications
Source: High Throughput. 2019 Jun 3;8(2):15. doi: 10.3390/ht8020015 (PMC6631403; doi:10.3390/ht8020015)
Supplement: Supplementary file 1 [file high-throughput-08-00015-s001.pdf]

# Supplementary Materials: Development and optimization of a miniaturized western blot-based screening platform to identify regulators of post-translational modifications

**Florencia Villafañez, Vanesa Gottifredi and Gastón Soria**

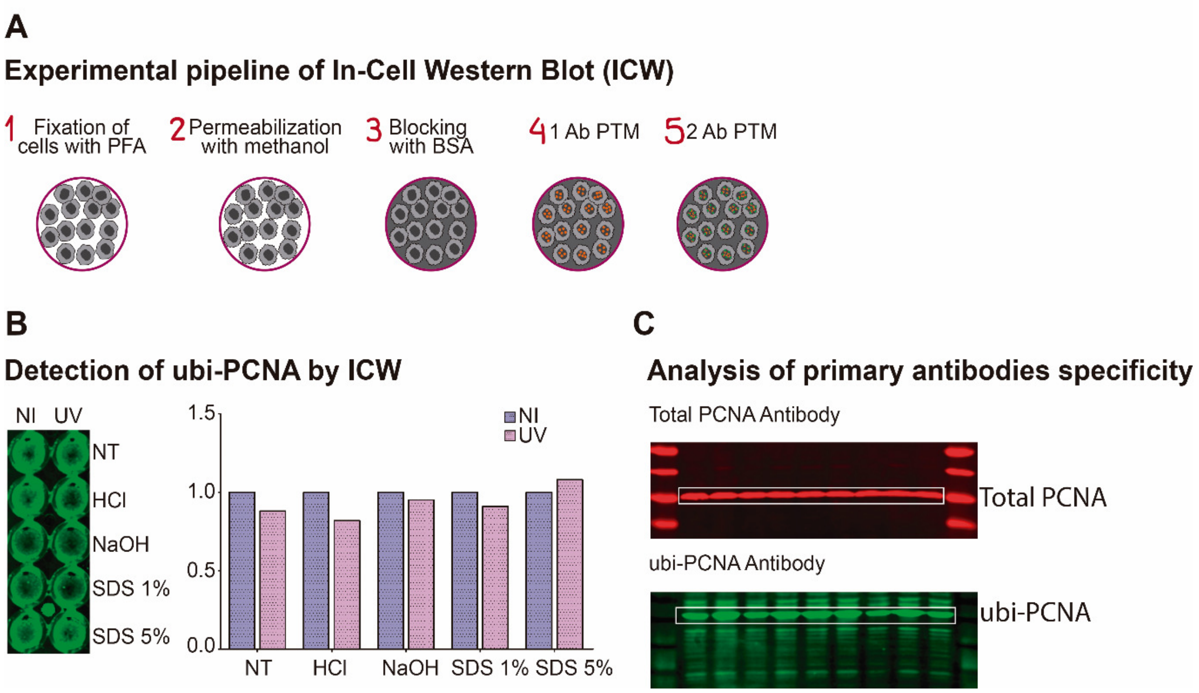


**Figure S1.** Experimental analysis of technique IC-WB for screening purposes. (**A**) General experimental pipeline for technique IC-WB; (**B**) Representative image depicting the detection of ubi-PCNA by IC-WB and quantification of fold induction; (**C**) Representative image of a WB to assess specificity of primary antibodies used in IC-WB. .
